# Supplementary material for: Automating the assessment of biofouling in images using expert agreement as a gold standard
Source: Sci Rep. 2021 Feb 2;11:2739. doi: 10.1038/s41598-021-81011-2 (PMC7854599; doi:10.1038/s41598-021-81011-2)
Supplement: Supplementary file 1 — Supplementary Information. [file 41598_2021_81011_MOESM1_ESM.pdf]

Automating the assessment of biofouling in images using expert agreement as a gold standard: Supporting Information

Evelyn J. Mannix[1,*], Susan Wei[2], Bartholomew A. Woodham[3], Peter Wilkinson[3], and Andrew P. Robinson[1]

[1]Centre of Excellence for Biosecurity Risk Analysis, The University of Melbourne, Melbourne, Australia
[2]School of Mathematics And Statistics, The University of Melbourne, Melbourne, Australia
[3]Biosecurity Animal Division, Department of Agriculture, Water and the Environment, Canberra, Australia

1 Network fitting

When training a convolutional neural network, as in most machine learning tasks, there are a number of key choices to make – the network architecture, the optimiser, the loss function, other hyperparameters, and the evaluation metric. In this section we examine various choices for each of these, taking as inspiration the design process of the Kaggle APTOS 2019 Blindness Detection competition[1]. We have relied heavily on the neural network library `PyTorch`[2] in `Python` throughout this project.

1.1 Loss and performance metrics

The performance metric and model loss are similar concepts but serve different purposes. The model loss is a score function used to train the network, and it needs to be differentiable so that the gradient can be used to optimise the model weights. The performance metric is used to choose the best performing epoch when training the model and selecting the best performing components and architectures.

For multi-class (K classes) classification a common performance metric is accuracy (A), which is given by the total proportion of classes correctly labelled (C) divided by the total number of predictions made (N)

$$A = \frac{C}{N} \tag{1}$$

and the model is trained using the categorical cross entropy loss, given by

$$\text{CELoss} = -\log\left(f(s)_t\right) \tag{2}$$

where the vector s is the raw output of the network, with the same number of elements as categories being predicted, t is the index of the correct class, and f is the softmax activation function. This calculates the probability the network thinks the image is actually of class t

$$f(s)_t = \frac{\exp^{s_t}}{\sum_i^K \exp^{s_i}} \tag{3}$$

However, in this formulation the distance between all classes is equal and this does not reflect the nature of our problem. Mistaking class zero as class two or vice versa is worse than mistaking class zero as class one. We will consider the following closely-related loss functions

$$\text{MSEloss} = (s-t)^2\,\alpha_t \tag{4}$$

$$\text{SmoothL1Loss} = \begin{cases} \frac{1}{2}(s-t)^2\,\alpha_t & \text{abs}(s-t) < 1 \\ \text{abs}(s-t)\,\alpha_t - \frac{1}{2} & \text{abs}(s-t) \geq 1 \end{cases} \tag{5}$$

where s is the raw output of the network, now a single number rather than a vector, and t is the index of the target class assuming they are placed in ascending order (SLoF 0 = 0, SLoF 1 = 1 and so on). The shape of both these functions is shown in Figure 1.

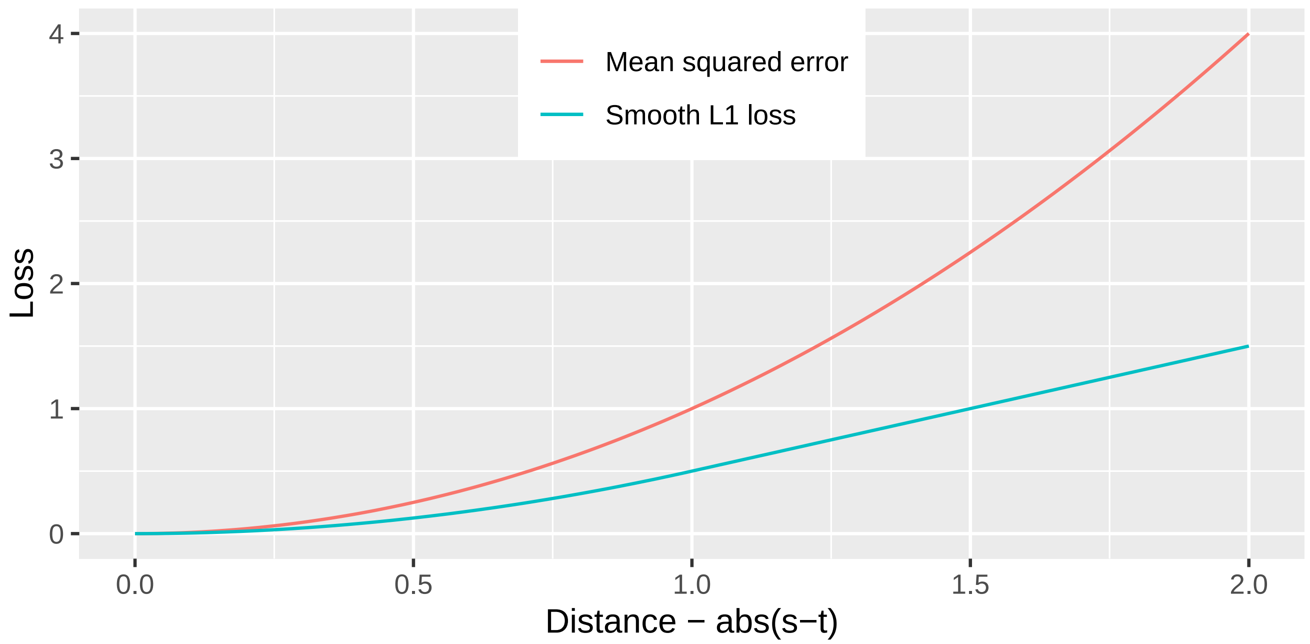

Figure 1. Loss functions used in model training.

These functions capture the ordinal nature of our labels, but the MSE loss function places a higher penalty on larger errors. We have also added a class weighting, α_t, which is given by

$$\alpha_t = \frac{N}{N_t} \tag{6}$$

where N_t is the number of images in class t. The classes in our dataset set were highly imbalanced, with significantly more SLoF 0 images than SLoF 2 images, so without this weighting we would optimise models that are biased to predicting an image is SLoF 0. If we knew the likely distribution of classes for our real-world use case it might be desirable to build a biased model, but this is currently unknown.

The APTOS competition used Cohen's Kappa with quadratic weights as their performance metric[3]. This requires the raw model output s to be thresholded into classes, and this was done by most teams by using thresholds of 0.5, 1.5, 2.5 and so on. For our purpose, this thresholding step is a decision that needs to be made after the model is trained to obtain the desired recall or sensitivity, as the consequences of mislabelling one class for another are asymmetric. Predicting a SLoF 0 image is SLoF 2 would be an acceptable mistake, but predicting a SLoF 2 image as SLoF 0 might be less so.

Instead we have chosen to use the average precision metric, which is calculated from a precision-recall curve. In our case we have two thresholds that need to be defined, which we split into images with a high biosecurity risk (SLoF $= 2$) and images with biofouling present (SLoF ≥ 2), and for our performance metric we take the mean of the average precision for these two binary classification problems.

1.2 Optimizers

The optimizer is responsible for taking the gradient of the loss and using it to update the network weights. Given a set of network weights θ and a cost function to optimize given by E

$$E(\theta) = J(\theta) \tag{7}$$

we can update θ using the gradient of E to find the optimal θ. This is called gradient descent, and each subsequent θ is given by

$$\theta_{i+1} = \theta_i - \eta \nabla_{\theta_i} E(\theta_i) \tag{8}$$

where η is our learning rate that determines how large the steps we take are, and θ_{i+1} is our new set of updated weights. Stochastic gradient descent (SGD) is a simple and widely used form of gradient descent. The weights θ are updated after each batch of images, which are randomly chosen without replacement from the full dataset. This random grouping of images

provides stochasticity and introduces a batch size parameter. A training epoch refers to one full iteration through the entire dataset.

SGD also adds momentum and weight decay as additional features. Momentum averages new weight updates with previous weight updates, for example if V_1 is the first weight update then we would proceed via

$$V_1 = \nabla_{\theta_1} E(\theta_1) \tag{9}$$
$$V_2 = \beta V_1 + (1-\beta)\nabla_{\theta_2} E(\theta_2) \tag{10}$$

where β is our momentum parameter. This can help improve the convergence rate, and a value between 0.9-0.99 is often suggested[4].

Weight decay penalises large weights, and is used to help stop the model from over fitting to the training data. This is done by modifying the original cost function. Previously our cost function was just given by the loss, but we can also add the weights to it to minimize their size

$$E(\theta) = J(\theta) + \frac{\lambda}{2}||\theta||^2 \tag{11}$$

where λ is the size of weight decay that we desire, and we can continue as above.

In addition to SGD, we also consider other optimisers including Adaptive Moment Estimation (Adam)[5], Rectified Adam (RAdam)[6] and Adam with a corrected weight decay algorithm (AdamW)[7]. These optimisers are similar to SGD, but do not have a momentum parameter. All of these methods were explored to determine which provided the best performance on our dataset.

1.3 Learning rate schedule

If only a constant learning rate is used, the network will reach a ceiling accuracy and be unable to fine-tune further. For this reason, it is helpful to reduce the learning rate as training progresses to obtain better results. We consider three different approaches, a multi step-learning rate schedule, a one-cycle schedule[4] and a cosine annealing schedule[8]. These learning rate schedules are shown in Figure 2, where we have chosen a initial maximum learning rate of 10^{-3} and minimum of 10^{-5}. For the one-cycle schedule, there is also a second minimum that is used in the fine-tuning period of 10^{-6}. The optimal learning rate and other parameters are explored further in Section 1.6.

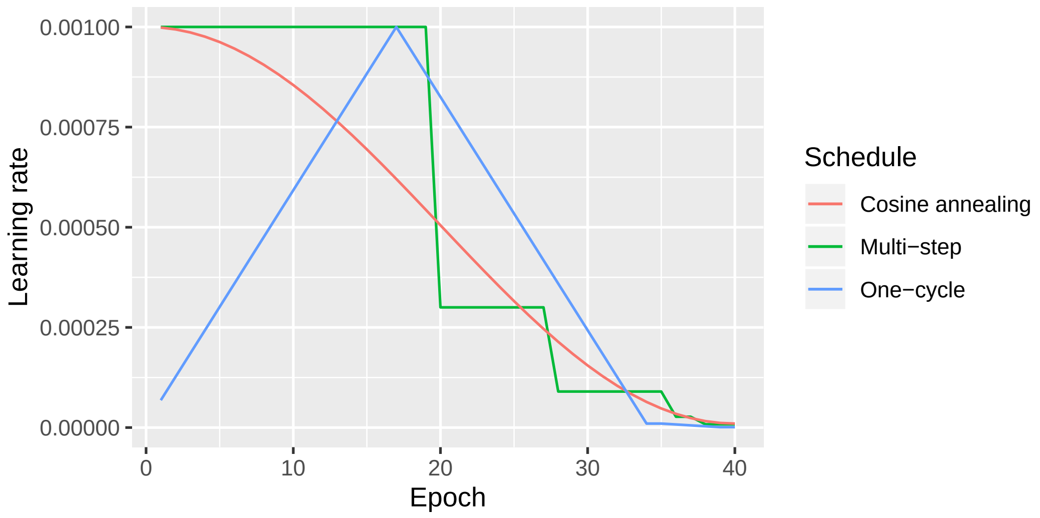

Figure 2. Learning rate schedules used in model training.

1.4 Image augmentation

Image augmentations play a key role in preventing the model from overfitting to the training data, and help improve how the model generalises to the testing and validation sets by varying features in the image that do not change its class, such as image orientation, brightness, contrast and so on. We adapted two image augmentation sequences from high scoring submissions in the APTOS competition that published their code on GitHub. These augmentations involved transforming the colour, brightness

and contrast of the image, rotating, flipping and cropping. From their original size (args.img_dim) both of these augmentation schemes crop images to a new extent (args.img_crop), which was always taken as an eighth smaller . For instance, images of size 256×256 were cropped to 224×224, while images of size 512×512 were cropped to 448×448. When the models were being evaluated, the images would be resized to have the same dimensions as the cropped training data. The code for the two image augmentations tested are shown below. The first used the `PyTorch` image augmentation library, while the second used `albumentations`. Sample transformed images are also shown in Figure 3.

Source Code 1. Simple image transform[9]

```
transform = transforms.Compose([
  transforms.RandomAffine(
      degrees=(-180, 180),
      translate=None,
      scale=(0.8889, 1.0),
      shear=(-36, 36)
  ),
  transforms.CenterCrop(args.img_crop),
  transforms.RandomHorizontalFlip(p=0.5),
  transforms.RandomVerticalFlip(p=0.5),
  transforms.ColorJitter( contrast=(0.9, 1.1) ),
  transforms.ToTensor(),
  transforms.Normalize([0.485, 0.456, 0.406], [0.229, 0.224, 0.225])

```

Source Code 2. Complex image transform[10]

```
transform = A.Compose([
  A.OneOf([
      A.ShiftScaleRotate(shift_limit=0.05, scale_limit=0.1,
                         rotate_limit=15,
                         border_mode=cv2.BORDER_CONSTANT, value=0),
      A.OpticalDistortion(distort_limit=0.11, shift_limit=0.15,
                          border_mode=cv2.BORDER_CONSTANT,
                          value=0),
      A.NoOp()
  ]),
  A.RandomSizedCrop(min_max_height=(int(args.img_dim * 0.75), args.img_dim),
                    height=args.img_crop,
                    width=args.img_crop, p=0.3),
  A.Resize(height=args.img_crop, width=args.img_crop),
  A.OneOf([
      A.RandomBrightnessContrast(brightness_limit=0.5,
                                 contrast_limit=0.4),
      IndependentRandomBrightnessContrast(brightness_limit=0.25,
                                          contrast_limit=0.24),
      A.RandomGamma(gamma_limit=(50, 150)),
      A.NoOp()
  ]),
  A.OneOf([
      FancyPCA(alpha_std=4),
      A.RGBShift(r_shift_limit=20, b_shift_limit=15, g_shift_limit=15),
      A.HueSaturationValue(hue_shift_limit=5,
                           sat_shift_limit=5),
      A.NoOp()
```

```
    ]),
    A.OneOf([
        ChannelIndependentCLAHE(p=0.5),
        A.CLAHE(),
        A.NoOp()
    ]),
    A.HorizontalFlip(p=0.5),
    A.VerticalFlip(p=0.5),
    A.Normalize(mean=[0.485, 0.456, 0.406], std=[0.229, 0.224, 0.225]),
    pyt.ToTensorV2()
])
```

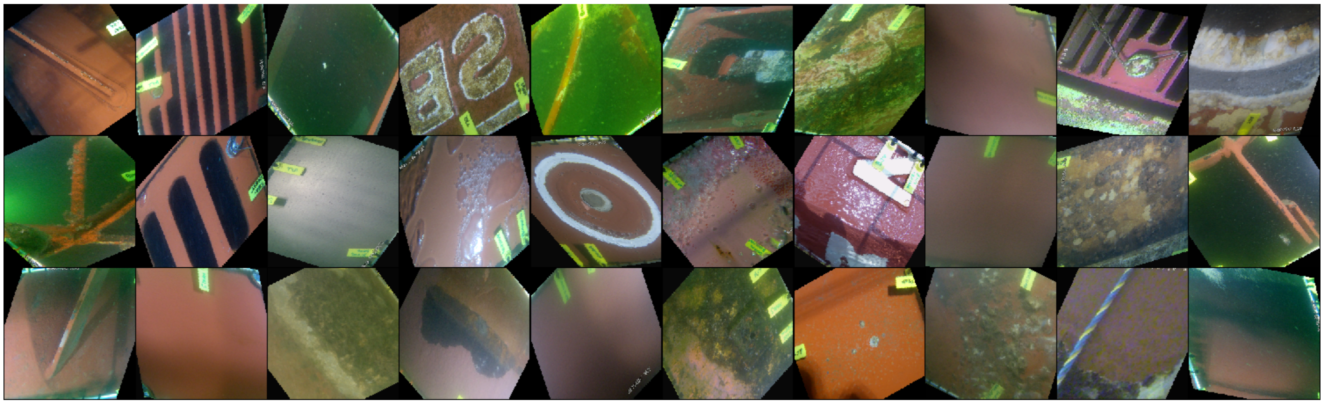

(a) Simple image transform

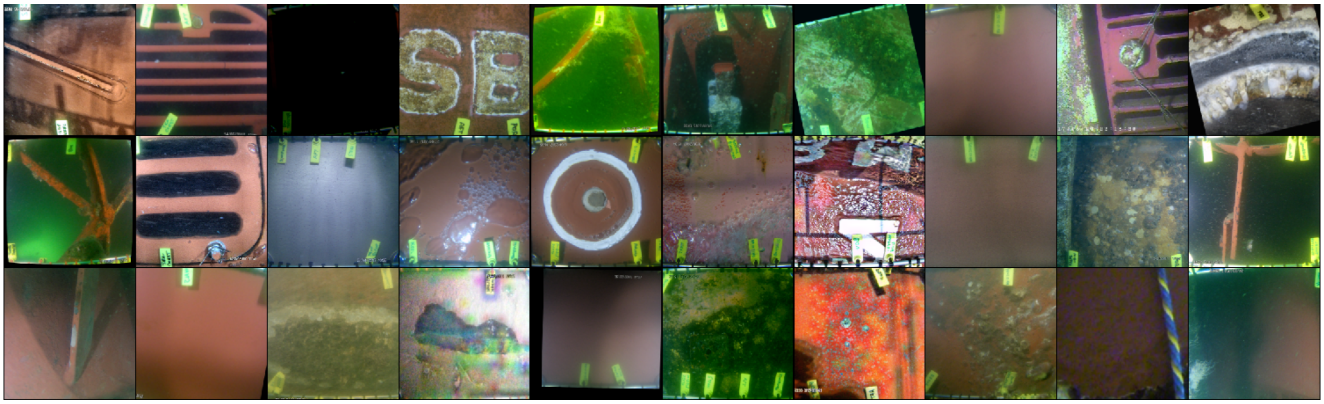

(b) Complex image transform

Figure 3. Examples of image transformations considered in model training.

1.5 Input image size

Images were input into the networks as a quarter of their size after pre-processing (256 by 256 pixels). The impact of using the full-size images (512 by 512 pixels) on model performance was also explored, but as this was quite computationally expensive it was only considered after other model training components had been selected. The impact of this change in resolution is shown for an example image in Figure 4.

1.6 Optimizer parameters

Before training networks and comparing model fits we need to choose parameters for the optimizer. These include the learning rate, batch-size, weight decay, and momentum if applicable. We initially conducted a learning rate test[11] to determine the range of learning rates under which training is stable. In this process, the learning rate was progressively increased from a low value and the validation loss was plotted. For this experiment we used the resnet18 architecture, SGD as the optimizer, weighted

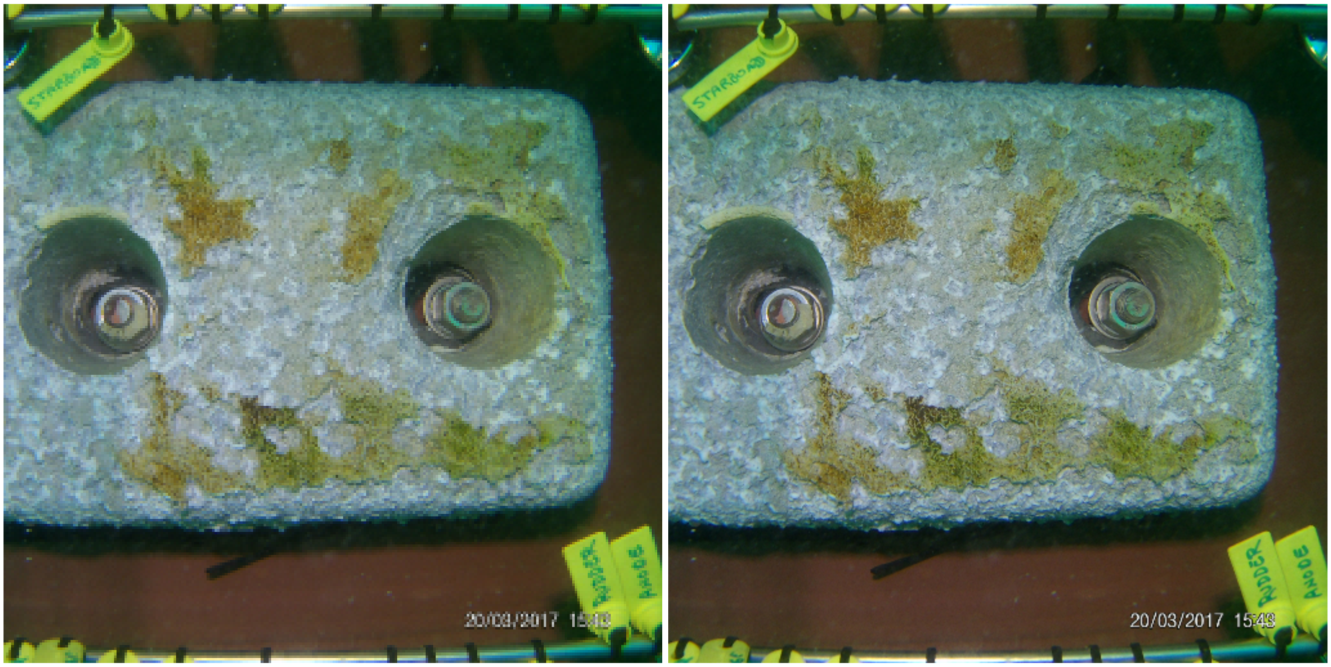

(a) 256x256 **(b)** 512x512

Figure 4. Impact of image size on image quality.

MSE loss, the simple set of image transforms, momentum of 0.9, weight decay of 10^{-4} and a batch-size of 32. The learning rate test results in Figure 5 show that after the learning rate increases past 10^{-3} training becomes unstable, and around 10^{-2} the loss increases sharply. This is consistent with the learning rates used in the APTOS competition, but fine tuning the learning rate and other parameters may still offer some improvement.

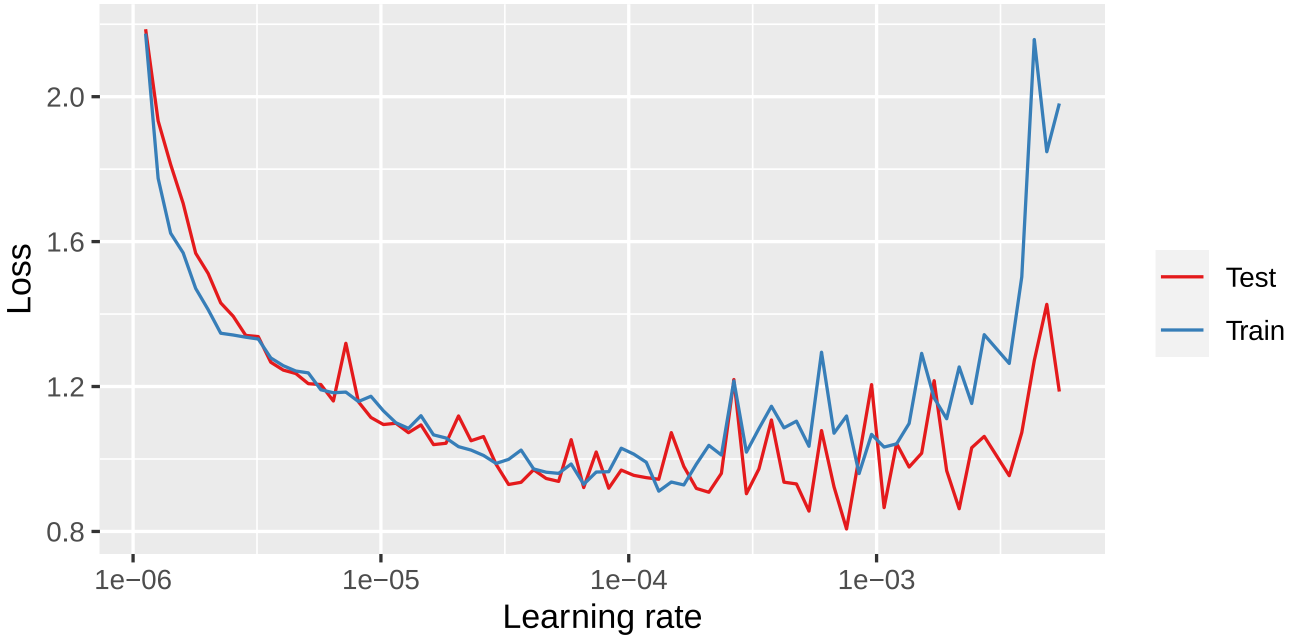

Figure 5. Learning rate test.

To explore this idea, we sampled parameters using the same base model but testing different combinations of batch-size,

weight decay, momentum and learning rate for short runs of 10 epochs. We considered batch sizes of 32 and 64 images, momentum between 0 and 0.99, and sampling on a log scale, weight decay between 10^{-6} and 10^{-2} and a learning rate between 10^{-5} and 10^{-2}. We sampled the momentum, weight decay and learning rate space using a Sobol sequence to obtain more even coverage[12], while trying all sets of these parameters for the different batch sizes. The best performing short runs are shown in Table 1. The only clear pattern was that a learning rate between 10^{-3} and 10^{-4} performed better, while momentum, batch sizes and weight decay values varied. We describe the spread in average precision between each crossfold with the standard deviation, which suggests between all top performing sets of parameters there is not a significant difference. The best sets of parameters for each batch size were explored with the other model components, in addition to our base set of parameters.

Table 1. Best short runs of 10 epochs obtained by sampling learning rate, weight decay, batch size and momentum parameters. For we ran five-fold stratified cross validation, where the best epoch from each was picked for comparison. The mean metric is the mean average precision on the validation set of each fold, and metric standard deviation is taken from the five crossfolds.

Learning rate (log10)	Weight decay (log10)	Momentum	Batch size	Mean metric	Metric SD
-3.22	-2.84	0.79	32	0.632	0.025
-3.03	-4.59	0.73	32	0.630	0.013
-3.78	-3.59	0.97	64	0.629	0.011
-2.98	-5.78	0.28	32	0.628	0.021
-3.03	-4.59	0.73	64	0.626	0.020
-3.26	-5.15	0.62	32	0.623	0.024
-3.22	-2.84	0.79	64	0.622	0.010
-3.31	-4.47	0.39	32	0.621	0.016
-2.94	-3.97	0.51	64	0.620	0.028
-3.12	-2.22	0.08	32	0.619	0.028

2 Comparing model fits

To find the most optimal set of training components we trained a resnet18 model with different combinations using five-fold stratified cross-validation. As described in the text we considered two different loss functions, MSE loss and smooth L1 loss, and also tested four different optimizers, including SGD, Adam[5], AdamW[7] and RAdam[6]. We tried the three sets of optimizer hyperparameters, three different learning rate schedules and two different image augmentation pipelines as described above. This resulted in 144 different models, of which the five best performing are shown in Table 2. We chose the top performing model components here to test with larger neural networks, noting that the difference between the top five models is not statistically significant.

Table 2. Best five models using the average precision metric. The mean metric is the mean average precision on the validation set of each fold, and metric standard deviation is taken from the five crossfolds.

Model	Hyperparam.	Loss	Optimizer	LR Schedule	Augmentations	Image size	Mean metric	Metric SD
resnet18	Tuned2	SL1	AdamW	MultiStep	Complex	256x256	0.710	0.030
resnet18	Tuned2	SL1	Adam	CosAnnealing	Complex	256x256	0.710	0.027
resnet18	Tuned2	MSE	AdamW	CosAnnealing	Complex	256x256	0.709	0.026
resnet18	Tuned2	MSE	Adam	OneCycle	Complex	256x256	0.705	0.021
resnet18	Tuned1	MSE	RAdam	CosAnnealing	Complex	256x256	0.704	0.028

The next step was to explore the impact of increasing the size of the images, and test the performance of larger networks. The results for the best models are shown in Table 3 using the networks described in the main text. These had improved perforance compared to the best performing models in Table 2.

Table 3. Best five larger models using the average precision metric. The same model components are used as in the best model in Table 2, with the exception of image size. The mean metric is the mean average precision on the validation set of each fold, and metric standard deviation is taken from the five crossfolds.

Model	Image size	Mean metric	Metric SD
se_resnext101_32x4d	512x512	0.754	0.024
se_resnext50_32x4d	512x512	0.751	0.029
inceptionresnetv2	512x512	0.750	0.025
efficientnet-b4	512x512	0.749	0.021
inceptionv4	512x512	0.747	0.024

2.1 Model ensembling

Model ensembling is a technique where the results from multiple trained networks are blended. There are numerous ways of doing this, up to building a statistical model that assists in the post-processing of model outputs. Here we explore the simplest approach, blending them by taking the average raw score[13]. We have identified the best ensembles by combining all of the models using the same image size with each other, and the top five performers are shown in Table 4. Combining the models in this way leads to a significant performance improvement over using just one model.

Table 4. Best five ensembles of larger models using the average precision metric. The mean metric is the mean average precision on the validation set of each fold, and metric standard deviation is taken from the five crossfolds.

Model	Image size	Mean metric	Metric SD
enb4,enb5,irv2,iv4,r18,sr101_32x4d,sr50_32x4d	512x512	0.796	0.023
enb5,irv2,iv4,r18,sr101_32x4d,sr50_32x4d	512x512	0.796	0.023
enb3,enb5,irv2,iv4,r18,sr101_32x4d,sr50_32x4d	512x512	0.796	0.022
enb3,enb4,enb5,irv2,iv4,r18,sr101_32x4d,sr50_32x4d	512x512	0.796	0.023
enb3,enb5,irv2,iv4,r18,sr101_32x4d	512x512	0.795	0.021

The top performing model in Table 4 was chosen as our best model, and is the one used in the results section of the paper. When applying the model, we use all crossfolds and average the result. This means our final model is actually made up of thirty different neural networks, with five crossfolds for each of the six models being ensembled above.

3 Annotation Interface and Examples

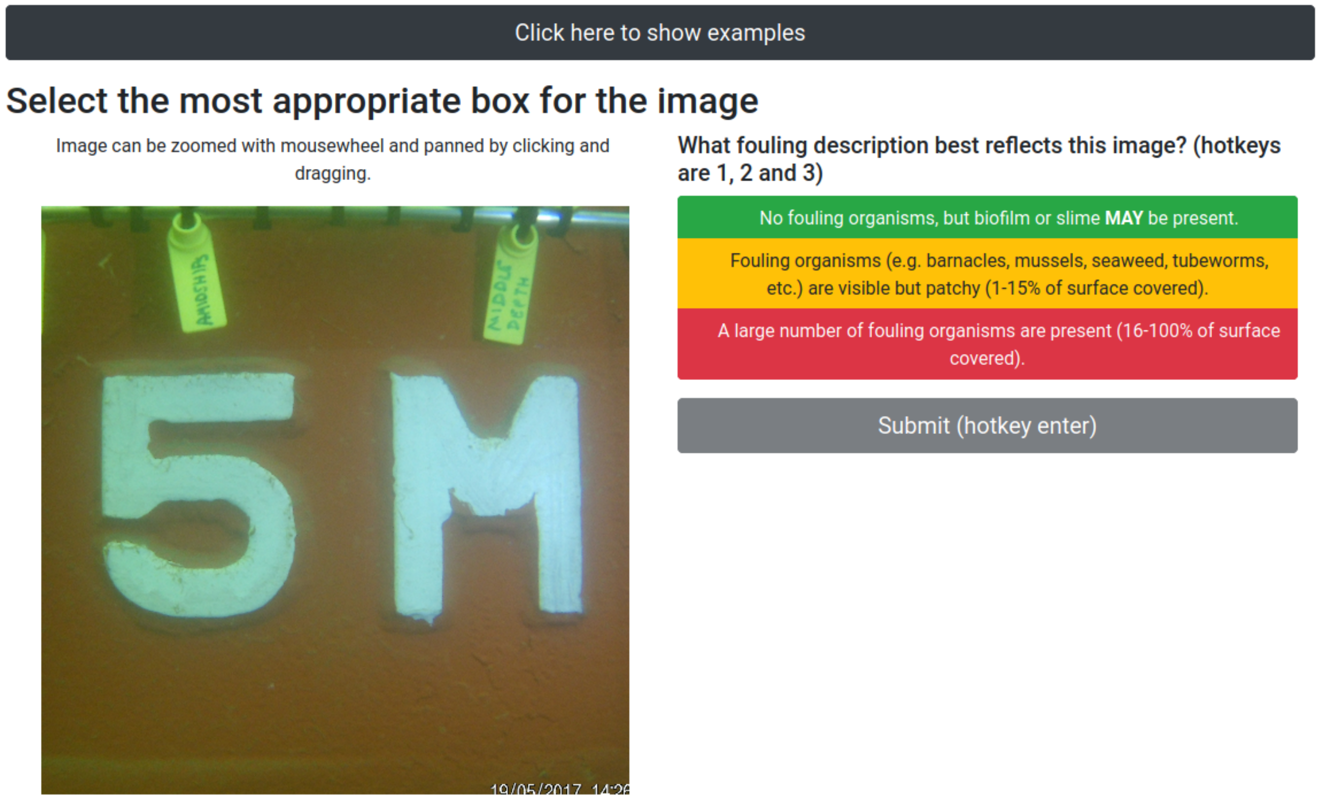

Figure 6. Interface used by experts for grading images. The examples provided within the interface for each category, under the "Click here to show examples" button, are given on the next page.

(a) Simplified Level of Fouling 0: No fouling organisms, but biofilm or slime MAY be present. Also keep in mind that rust is NOT fouling.

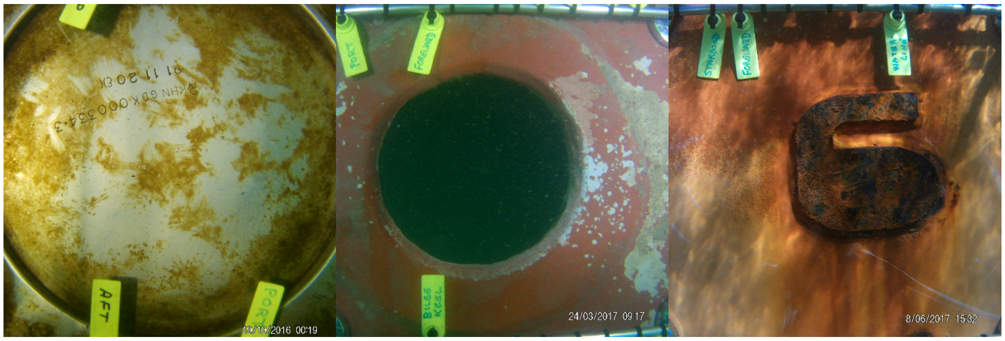

(b) Simplified Level of Fouling 1: Fouling organisms (e.g. barnacles, mussels, seaweed, tubeworms, etc.) are visible but patchy (1-15% of surface covered). If there is ATLEAST one organism present, then use this category.

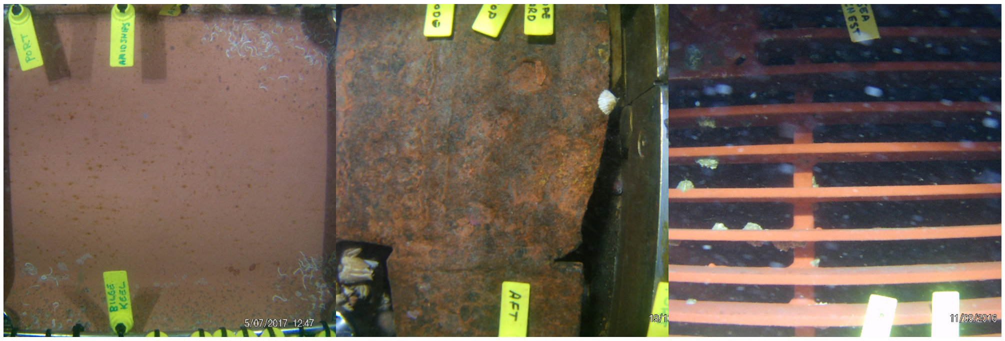

(c) Simplified Level of Fouling 2: A large number of fouling organisms are present (16-100% of surface covered).

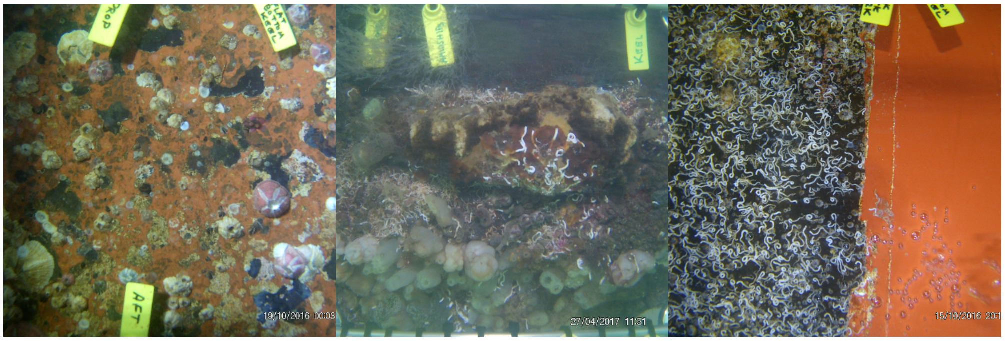

Figure 7. Example images.

References

1. Kaggle. APTOS 2019 Blindness Detection. https://www.kaggle.com/c/aptos2019-blindness-detection (2020). Accessed: 2020-05-07.
2. Paszke, A. *et al.* PyTorch: An imperative style, high-performance deep learning library. In *Advances in Neural Information Processing Systems 32*, 8024–8035 (Curran Associates, Inc., 2019).
3. scikit-learn. cohen_kappa_score. https://scikit-learn.org/stable/modules/generated/sklearn.metrics.cohen_kappa_score.html (2020). Accessed: 2020-05-07.
4. Smith, L. N. A disciplined approach to neural network hyper-parameters: Part 1–learning rate, batch size, momentum, and weight decay. *arXiv preprint arXiv:1803.09820* (2018).
5. Kingma, D. P. & Ba, J. Adam: A method for stochastic optimization. *arXiv preprint arXiv:1412.6980* (2014).
6. Liu, L. *et al.* On the variance of the adaptive learning rate and beyond. *arXiv preprint arXiv:1908.03265* (2019).
7. Loshchilov, I. & Hutter, F. Decoupled weight decay regularization. *arXiv preprint arXiv:1711.05101* (2017).
8. Loshchilov, I. & Hutter, F. Sgdr: Stochastic gradient descent with warm restarts. *arXiv preprint arXiv:1608.03983* (2016).
9. 4ui_iurz. 11th place solution. https://www.kaggle.com/c/aptos2019-blindness-detection/discussion/107958 (2020). Accessed: 2020-06-26.
10. Khvedchenya, E. 7th place solution. https://www.kaggle.com/c/aptos2019-blindness-detection/discussion/108058 (2020). Accessed: 2020-06-26.
11. Smith, L. N. Cyclical learning rates for training neural networks. In *2017 IEEE Winter Conference on Applications of Computer Vision (WACV)*, 464–472 (IEEE, 2017).
12. Atanassov, E. I. A new efficient algorithm for generating the scrambled Sobol sequence. In *International Conference on Numerical Methods and Applications*, 83–90 (Springer, 2002).
13. Norouzzadeh, M. S. *et al.* Automatically identifying, counting, and describing wild animals in camera-trap images with deep learning. *Proc. Natl. Acad. Sci.* **115**, E5716–E5725 (2018).
